# Differential Hematotoxic Activity of Southeast Asian Pit Viper Venoms: The Cross-Neutralizing Effect of Available Antivenoms

**DOI:** 10.3390/medsci14020199

**Published:** 2026-04-14

**Authors:** Dollapak Apipongrat, Muhamad Rusdi Ahmad Rusmili, Kornkanok Thapanasopon, Khatsophon Poonaya, Wittawat Chantkran, Janeyuth Chaisakul

**Affiliations:** 1 Department of Pathology, Phramongkutklao College of Medicine, Bangkok 10400, Thailand; dollapak.a@gmail.com (D.A.); chantkran@yahoo.com (W.C.); 2 Department of Basic Medical Sciences, Kulliyyah of Pharmacy, IIUM Centre for Natural and Health Sciences Kuantan Campus, International Islamic University Malaysia, Kuantan 25200, Pahang Darul Makmur, Malaysia; rusdirusmili@iium.edu.my; 3 Division of Academic Affairs, Phramongkutklao College of Medicine, Bangkok 10400, Thailand; std6447002@pcm.ac.th (K.T.); std6447008@pcm.ac.th (K.P.); 4 Department of Pharmacology, Phramongkutklao College of Medicine, Bangkok 10400, Thailand

**Keywords:** antivenom, venom, snake, coagulation, platelet aggregation

## Abstract

**Background/Objectives:** Pit vipers (subfamily Crotalinae) are responsible for a large proportion of snakebite envenoming cases in Southeast Asia. Envenomation by these snakes commonly causes hematotoxic effects, including platelet dysfunction and coagulation disturbances. Although antivenom remains the mainstay of treatment, species-specific antivenoms are not available for several regional pit viper species. This study evaluated the hematotoxic activities of selected Southeast Asian pit viper venoms and the cross-neutralizing capacity of commercially available antivenoms. **Methods:** Venoms from five medically important pit viper species—*Calloselasma rhodostoma*, *Trimeresurus albolabris*, *T. hageni*, *T. purpureomaculatus*, and *Tropidolaemus wagleri*—were tested. Washed platelets and platelet-poor plasma obtained from healthy individuals (*n* = 10) were used to assess venom-induced platelet aggregation and coagulation, respectively. The neutralizing effects of three antivenoms including hemato polyvalent antivenom (HPAV), *T. albolabris* antivenom (TAAV), and *C. rhodostoma* antivenom (CRAV)—were examined in vitro. **Results:** All tested venoms induced in vitro platelet aggregation (%Max > 50%) and promoted plasma coagulation. At the manufacturer-recommended concentration, TAAV significantly cross-neutralized the hematotoxic effects of *T. purpureomaculatus* and *T. hageni* venoms (*p* < 0.0001) but failed to neutralize coagulation induced by *T. wagleri*. CRAV showed no cross-neutralization against arboreal pit viper venoms. In contrast, HPAV strongly inhibited platelet aggregation and coagulation induced by all tested venoms (*p* < 0.0001). **Conclusions:** These findings highlight the limited cross-neutralization capacity of monovalent antivenoms against arboreal pit viper hematotoxicity. In contrast, HPAV demonstrated broad cross-neutralizing activity and may represent a practical therapeutic option for Southeast Asian pit viper envenoming when species-specific antivenoms are unavailable.

## 1. Introduction

Snakes of the family Viperidae are classified into two subfamilies: the typical vipers (subfamily Viperinae) and the pit vipers (subfamily Crotalinae). These snakes possess relatively long, hollow fangs that normally lie folded against the upper jaw but are erected when the snake strikes [1]. Members of the subfamily Crotalinae have a specialized sensory structure known as the loreal pit organ, which enables them to detect the body heat of warm-blooded prey, particularly rodents and birds. This organ is located between the nostril and the eye [2,3]. Pit vipers are commonly encountered venomous snakes and are responsible for a substantial proportion of snakebite envenoming cases. This high incidence is largely attributed to the expansion of agricultural and residential areas into regions overlapping with snake habitats, combined with limited access to adequate medical care [4,5]. *Calloselasma rhodostoma* (Malayan pit viper) and *Trimeresurus albolabris* (white-lipped pit viper) are recognized as medically significant venomous pit vipers (Category 1) causing the highest frequency of envenoming incidents and resulting in the high mortality and morbidity rates in Thailand [6]. The hematotoxic effects caused by pit viper envenomation commonly include local blistering, tissue necrosis, shock resulting from spontaneous systemic bleeding, fibrinolysis, thrombocytopenia, abnormal blood pressure, and leukocytosis [7,8,9]. The underlying mechanisms of these pathological effects have been attributed to the presence of hematotoxic proteins and enzymes, including snake venom metalloproteinases (SVMPs), phospholipase A_2_ (PLA_2_), snake venom serine proteases (SVSPs), and L-amino acid oxidase (LAAO) [10,11].

The Malayan pit viper accounted for a high incidence of snakebite cases during the decade between 2011 and 2021, particularly in the southern regions of Thailand [6,9]. In contrast, most pit viper envenoming cases in Bangkok were caused by green pit vipers (*Trimeresurus* spp.) [12]. In fact, the *Trimeresurus* pit viper complex comprises more than 40 recognized species, many of which are venomous and medically important. Notable examples include *T. albolabris* (white-lipped pit viper), *T. hageni* (Hagen’s pit viper), and *T. purpureomaculatus* (mangrove pit viper) [13]. In addition, species of the genus *Tropidolaemus* are among the causes of venomous snakebites in Southeast Asia. *Tropidolaemus wagleri* (the temple pit viper) venom typically results in local effects such as pain and swelling, as well as neurotoxic manifestations attributed to the presence of abundant neurotoxic peptides known as waglerins [14]. Unlike other pit vipers in the region, the venom of *T. wagleri* has been reported to exhibit weak pseudo-procoagulant activity, inducing fibrin clot formation with minimal overall anticoagulant effect. Nevertheless, a recent proteomic analysis demonstrated a high abundance of hematotoxic enzymes in *T. wagleri* venom, including SVSPs and LAAO [11]. Accordingly, additional studies are warranted to elucidate the potential hematotoxic effects of *T. wagleri* envenomation.

The administration of polyclonal antibodies, commonly referred to as antivenom, remains the most effective treatment for snakebite envenomation [15]. The antivenom works by binding to its respective complementary binding site on the surface of the toxins, forming a complex that changes the toxins’ molecular configuration, causing steric hindrance and rendering them unable to bind to their respective molecular target. This eventually prevents the toxic effects of the venoms [16]. However, specific antivenoms are not currently available for all snake species. Owing to interspecific variation in venom composition, the efficacy of available antivenoms can differ markedly among snake species and even among populations of the same species. In situations where species-specific antivenoms are unavailable, locally produced or imported antivenoms with demonstrated cross-neutralizing activity—defined as the ability to neutralize the toxic effects of venoms from related species not included in the original immunizing mixture—are often used as alternative treatments. Therefore, promoting the use of antivenoms with verified cross-neutralization capabilities represents a practical strategy to improve antivenom accessibility and enhance treatment outcomes [17].

In Thailand and some Southeast Asian countries, systemic snake envenomation is commonly treated with monovalent or polyvalent antivenoms produced by the Queen Saovabha Memorial Institute (QSMI) of the Thai Red Cross Society, Thailand. The QSMI manufactures three monospecific antivenoms targeting medically important vipers in Thailand: *Daboia siamensis* antivenom (DSAV), *C. rhodostoma* antivenom (CRAV), and *T. albolabris* antivenom (TAAV). In addition, QSMI produces a polyvalent formulation, the Hemato Polyvalent Snake Antivenom (HPAV), which is effective against the venoms of these three viper species [18]. Previous reports showed that many hetero-specific snake antivenoms demonstrated cross-neutralizing activity against venoms of other viper species in both in vivo [19] and in vitro [20] experiments. Consequently, administration of the QSMI monospecific antivenoms such as CRAV and TAAV has been considered a potential treatment option for envenomation by lesser-known pit viper species in Southeast Asia [21].

In this study, we aimed to evaluate the procoagulant activities, specifically platelet aggregation and coagulation, of venoms from five medically significant pit viper species found in Southeast Asia, including *C. rhodostoma*, *T. wagleri*, *T. hageni*, *T. albolabris*, and *T. purpureomaculatus*. In addition, the cross-neutralizing effects and efficacy of three commercially available snake antivenoms, TAAV, CRAV, and HPAV, on venom-induced platelet aggregation and coagulation were also investigated. This study provides insight into the mechanisms underlying hematotoxic outcomes following envenomation by Asian pit vipers and contributes to the development of improved treatment strategies.

## 2. Materials and Methods

### 2.1. Study Population and Plasma Sample Collection

Leftover citrated blood samples were obtained from 10 healthy individuals (6 males and 4 females, with a mean age ± SD of 27.4 ± 6.9 years) participating in a routine check-up program at the Special Hematology Laboratory, Division of Hematology, Department of Medicine, Phramongkutklao Hospital, Bangkok, Thailand, between 15 and 25 October 2024.

For routine evaluation, 10 mL of 3.2% sodium citrate blood was collected from each individual for coagulation testing (coagulogram, fibrinogen levels, and D-dimer) and processed directly in our laboratory. After completion of routine testing, only samples with a remaining volume greater than 9 mL were included in the study. This ensured sufficient volume for the preparation of washed platelets and platelet-poor plasma (PPP), as well as for subsequent experimental analyses. Samples with insufficient residual volume were excluded. To minimize pre-analytical variability, all samples were processed within 2 h of collection in accordance with standard laboratory procedures.

All included samples demonstrated normal baseline coagulation parameters, including prothrombin time (PT), activated partial thromboplastin time (aPTT), thrombin time (TT), fibrinogen levels, D-dimer, and platelet counts. Although some potentially relevant clinical information—such as smoking status, medication use, and hormonal therapy—was not available, all samples demonstrated normal platelet aggregation responses upon thrombin stimulation, confirming the functional integrity and suitability of the platelets used in this study.

### 2.2. Snake Venoms and Antivenoms

Lyophilized pooled snake venoms of *T. albolabris* and *T. purpureomaculatus* were purchased from Snake Farm of the QSMI, Thai Red Cross Society, Thailand. Venoms of *C. rhodostoma* and *T. hageni* were purchased from Latoxan Laboratory, France. The approval number for the use of animal venom is 2564-21-0073 (approval date: 13 June 2024). *T. wagleri* venom was kindly provided by Dr Muhamad Rusdi Ahmad Rusmili, International Islamic University Malaysia. The research permit for Malaysian snakes was provided by the Department of Wildlife and National Parks, Government of Malaysia (Permit No: HQ-00067-15-70; permission on 4 June 2018). *T. wagleri* was milked from specimens captured in northwest Peninsular Malaysia. The venom from three individuals from each species was extracted by allowing the snakes to bite plastic containers wrapped with parafilm. The specimens were milked three times with a time interval of three weeks between milking, before being released at the area of capture. Pools of venom from each species were then generated, frozen and freeze-dried. When required, lyophilized venom was weighed and reconstituted in phosphate-buffered saline (PBS), and its protein concentration was determined using a BCA protein assay (Pierce Biotechnology, Rockford, IL, USA).

A hemato polyvalent antivenom (HPAV, Lot no. HP00224) against the venom of *C. rhodostoma*, *T. albolabris* and *D. siamensis*, along with monovalent antivenoms specific to *T. albolabris* (TAAV, Lot no. TA00124) and *C. rhodostoma* (CRAV, Lot no. CR00523), was used in this study. All antivenoms were equine F(ab’)_2_-based preparations produced by the QSMI, Thai Red Cross Society, Thailand. Inhibition or neutralization assays using antivenoms were conducted according to the antivenin manufacturer’s ratio instructions; i.e., 1.0 mL of HPAV is capable of neutralizing 1.6 mg of *C. rhodostoma* venom, 0.7 mg of *T. albolabris* venom, and 0.6 mg of *D. siamensis* venom. Similarly, 1.0 mL of TAAV neutralizes 0.7 mg of *T. albolabris* venom, while 1.0 mL of CRAV neutralizes 1.6 mg of *C. rhodostoma* venom.

### 2.3. Plasma Sample and Washed Platelet Preparations

Plasma samples were prepared in accordance with the Clinical and Laboratory Standards Institute (CLSI) guidelines and recommendations [22,23]. Blood was collected into 3.2% sodium citrate evacuated polymer tubes (Vacutainer, Greiner Bio-One, Stonehouse, UK) at a 9:1 blood-to-anticoagulant ratio. The protocol for platelet isolation and preparation of washed platelets followed previously published methods [24,25]. Briefly, citrated blood from each individual was pre-warmed to 37 °C and centrifuged at 250× *g* for 15 min at room temperature. The resulting platelet-rich plasma (PRP) was carefully collected and incubated at 37 °C for 10 min. It was then centrifuged at 2200× *g* for 15 min to obtain PPP, which was transferred to 5 mL vial tubes and stored at −20 °C for subsequent clotting time-based analyses. The remaining platelet pellet was resuspended in 10 mL of Tyrode’s buffer (Sigma-Aldrich, St. Louis, MO, USA) and centrifuged at 2000× *g* for 5 min. The supernatant was discarded, and the washing step was repeated twice. In the final step, the washed platelet pellet was resuspended in 5 mL of Tyrode’s buffer. Platelet counts were performed using a Sysmex XN-9000 automated hematology analyzer (Sysmex Corporation, Kobe, Japan) and adjusted with the same buffer to achieve a final concentration of 300 × 10^9^/L.

### 2.4. Venom-Induced Platelet Aggregation Test

A venom-induced platelet aggregation test was conducted using light transmission aggregometry (LTA) on an AggRAM platelet aggregometer (Helena Biosciences, Gateshead, UK), operated under continuous magnetic stirring at 1000 rpm, a constant temperature of 37 °C, and a measurement duration of 10 min. Tyrode’s buffer served as the reference for 100% aggregation.

Venoms of *C. rhodostoma*, *T. wagleri*, *T. hageni*, *T.albolabris* and *T. purpureomaculatus* were diluted in distilled water to achieve a concentration of 1.0 mg/mL (0.05 mg of venom), and used as platelet agonists. Thrombin at 10 IU/mL was used as a positive control to assess the functional quality of the washed platelets.

The platelet aggregation protocol was as follows: 450 µL of washed platelets from each individual (*n* = 10) was added to a reaction cuvette containing a magnetic stir bar. The sample was pre-warmed at 37 °C for 5 min, after which 50 µL of the agonist was added to initiate the measurement. Percentage of maximum aggregation (%Max) and slope of the aggregation curve were recorded.

### 2.5. Platelet Aggregation Inhibition Assay Using Antivenom

To evaluate the inhibitory effect of the antivenoms on platelet aggregation, the volume required to neutralize 0.05 mg of snake venom was calculated. It was estimated that 72 µL of each antivenom was needed to neutralize the venoms of *T. wagleri*, *T. hageni*, *T. albolabris*, and *T. purpureomaculatus*, while only 32 µL of the antivenoms was sufficient to neutralize the venom of *C. rhodostoma*.

Each antivenom was added to washed platelets to achieve a total volume of 450 µL. For the venoms of *T. wagleri*, *T. hageni*, *T. albolabris*, and *T. purpureomaculatus*, 72 µL of antivenom was mixed with 378 µL of washed platelets. For *C. rhodostoma* venom, this consisted of 32 µL of antivenom and 418 µL of washed platelets. The mixture was incubated at 37 °C for 5 min before platelet aggregation was assessed according to the protocol described above. In this investigation, thrombin at 10 IU/mL (Dade^®^ Thrombin Reagent, Siemens Healthineers, Marburg, Germany) was used as a positive control to verify the functional integrity of the washed platelets. Additionally, normal saline solution (NSS) was used in place of antivenom as a baseline control for each individual. Platelet aggregation results from the baseline and from washed platelets treated with antivenom were compared individually. The percentage of inhibition (%inhibition) was calculated using the following equation:


$$\%inhibition=\frac{\left[(\%Max\;of\;baseline)\;-\;(\%Max\;of\;washed\;platelets\;trated\;with\;antivenom)\right]}{\%Max\;of\;baseline}\times 100$$

### 2.6. Venom-Induced Coagulation Test

The PPP samples from each healthy individual (*n* = 10) were used to evaluate venom-induced clotting time (CT). To compare the effects of venom on coagulation and determine the optimal concentration for each snake species, working venom solutions were prepared by diluting lyophilized venoms in DW to final concentrations of 0.25, 0.50, 0.75, 1.00, and 2.00 mg/mL. The CT of the venom-induced coagulation test was measured as follows: 100 µL of PPP was pre-warmed at 37 °C for 3 min, followed by the addition of 50 µL of the working venom solution to initiate clotting. The adapted test protocol was implemented using a Sysmex CS-2500 coagulation analyzer (Sysmex Corporation, Kobe, Japan).

### 2.7. Coagulation Inhibition Using Antivenom

To assess the inhibitory effect of antivenoms on venom-induced coagulation, a mixture of 60 µL of antivenom and 60 µL of PPP was incubated at 37 °C for 3 min, followed by the addition of 40 µL of a 1.0 mg/mL working venom solution to initiate clotting. For *C. rhodostoma* venom specifically, a mixture of 25 µL of antivenom, 35 µL of NSS and 60 µL of PPP was used, based on the neutralizing dose specified in the manufacturer’s instructions for HPAV and CRAV. Thrombin at 10 IU/mL was used as an unaffected control. The adapted test protocol was performed using the Sysmex CS-2500 coagulation analyzer. NSS was used in place of antivenom as an unaffected control for each individual. Baseline CTs induced by each venom and by thrombin were recorded. The percentage of CT prolongation (% prolongation) for each venom was subsequently calculated using the following equation:


$$\%prolongation=\frac{\left[(CT\;of\;PPP\;trated\;with\;antivenom)\;-\;(CT\;of\;basline)\right]}{CT\;of\;basline}\times 100$$

### 2.8. Statistical Analysis

Statistical analyses were conducted using IBM SPSS version 21 (IBM Corp., Armonk, NY, USA) and GraphPad Prism, version 9.0 (GraphPad Software, San Diego, CA, USA). The sample size was calculated based on the mean thrombin time derived from a locally validated cohort of healthy individuals (*n* = 120) used for reference interval establishment. Assuming a standard deviation (SD) of 0.16, a significance level (α) of 0.05, and a margin of error of 0.1, the minimum required sample size was estimated to be 9.83 and was rounded up to 10 samples. Continuous variables were expressed as mean ± standard deviation (SD) or median with interquartile range (IQR), as appropriate. Comparisons between two conditions were performed using paired Student’s *t*-test or the Wilcoxon signed-rank test, depending on data distribution. For comparisons among multiple conditions, one-way repeated-measures ANOVA followed by Tukey’s post hoc test or the Kruskal–Wallis test followed by Dunn’s post hoc test was used, as appropriate. Post hoc power analyses were conducted for the primary paired comparisons (α = 0.05) based on the observed effect sizes (Cohen’s d). Across all assays, most comparisons demonstrated high statistical power, ranging from approximately 80.3% to >99.9%, depending on the observed effect size. A *p*-value < 0.05 was considered statistically significant.

### 2.9. Reverse-Phase HPLC

Venoms were dissolved in 0.1% formic acid (Fisher Scientific, Geel, Belgium) in water at a final concentration of 1 mg/mL before being filtered using a 45 µm syringe filter. The supernatants (40 μL) were loaded into an AERIS XB-C18 3.6 μm C18 300 Å reverse-phase column (Phenomenex, Torrance, CA, USA) mounted on an Agilent 1260 Infinity semi-preparative high-pressure liquid chromatography system (Agilent Technologies, Santa Clara, CA, USA). The column was equilibrated with 0.1% formic acid in water (solution A) and the peaks were eluted from the column with 90% acetonitrile (Fisher Scientific, Seoul, Republic of Korea) in 0.1% formic acid in water (solution B) using the following gradient: 5% solution B from 0 to 5 min, 5–65% solution B from 5 to 50 min and 65–100% solution B from 50 to 60 min at a flow rate of 0.25 mL/min. The eluted peaks were monitored at 214 nm and analyzed using OpenLAB CDS Workstation (Agilent Technologies, Waldbronn, Mannheim, Germany).

## 3. Results

### 3.1. Venom Reverse-Phase High-Performance Liquid Chromatography (RP-HPLC)

**Figure 1:**
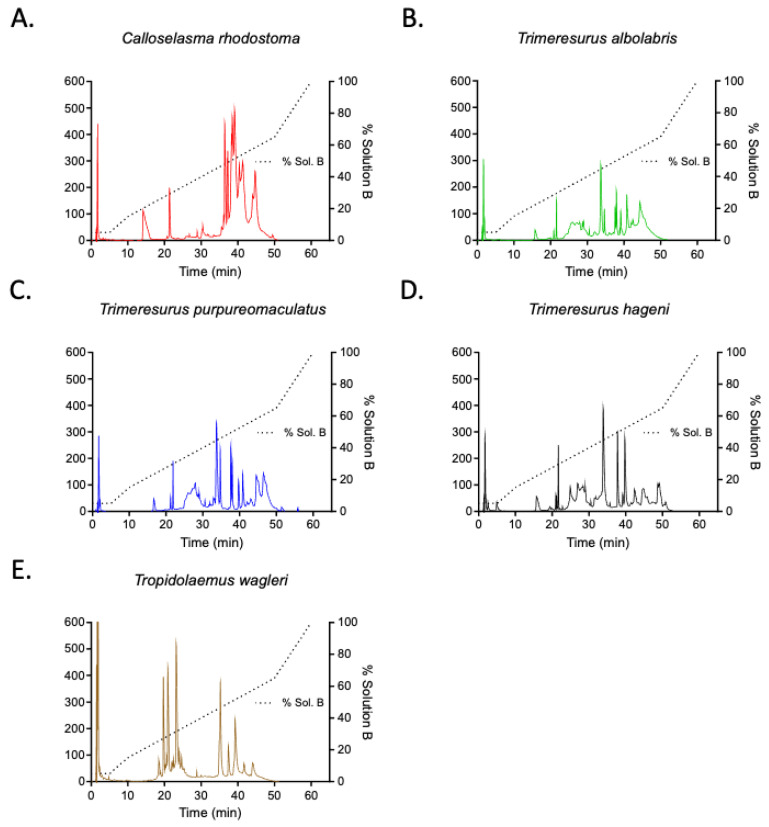
RP-HPLC chromatograms of (**A**) *C. rhodostoma* venom, (**B**) *T. albolabris* venom, (**C**) *T. purpureomaculatus* venom, (**D**) *T. hageni* venom and (**E**) *T. wagleri* venom, run with the same conditions on an AERIS XB-C18 3.6 μm C18 300 Å reverse-phase column, equilibrated with 0.1% formic acid in water (solution A); the peaks were eluted from the column with 90% acetonitrile in 0.1% formic acid in water (solution B) using the following gradient: 5% solution B from 0 to 5 min, 5–65% solution B from 5 to 50 min and 65–100% solution B from 50 to 60 min at a flow rate of 0.25 mL/min. The eluted peaks were monitored at 214 nm and analyzed using OpenLAB CDS Workstation.

RP-HPLC analysis of the venoms indicated marked differences in chromatogram profiles (Figure 1A–E). Approximately 22 peaks were eluted in the profile for *C. rhodostoma* venom (Figure 1A). *T. albolabris*, *T. purpuremaculatus* and *T. hageni* venoms exhibited 17–18-peak profiles (Figure 1B–D). *T. wagleri* venom displayed a 14-peak profile (Figure 1E).

### 3.2. Effects of Pit Viper Venoms on Human Platelet Function

**Figure 2:**
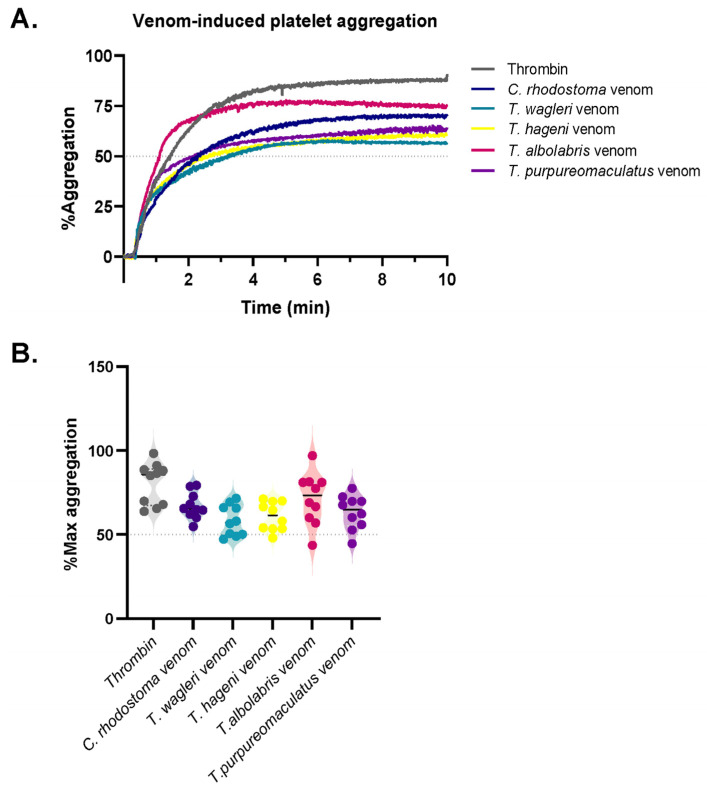
Venom-induced platelet aggregation. (**A**) Representative aggregation curves showing the mean platelet aggregation induced by thrombin and *C. rhodostoma*, *T. wagleri*, *T. hageni*, *T. albolabris*, and *T. purpureomaculatus* venoms. (**B**) Violin plots depicting the percentage of maximum platelet aggregation (%Max aggregation) induced by thrombin and each venom.

Washed platelets from 10 healthy individuals were used to assess venom-induced platelet aggregation. The mean aggregation curves for each venom are presented in Figure 1A. All snake venoms caused in vitro platelet aggregation, with a % Max aggregation exceeding 50%. At an equal venom concentration (1.0 mg/mL; 0.05 mg), *T. albolabris* venom caused the highest %Max aggregation (71.4 ± 15.3%) followed by the venoms of *C. rhodostoma* (67.3 ± 11.9%)*, T. purpureomaculatus* (63.3 ± 10.1%)*,* and *T. hageni* (61.7 ± 14.1%), while *T. wagleri* caused the least platelet aggregation with a %Max aggregation of 58.5 ± 9.1% (Figure 2).

To verify the functional integrity of the washed platelets, thrombin (10 IU/mL) was used as a positive control, yielding a mean ± SD %Max aggregation of 80.5 ± 12.4%, indicating preserved platelet function.

### 3.3. Neutralizing Effect of Antivenoms Against Venom-Induced Platelet Aggregation

**Figure 3:**
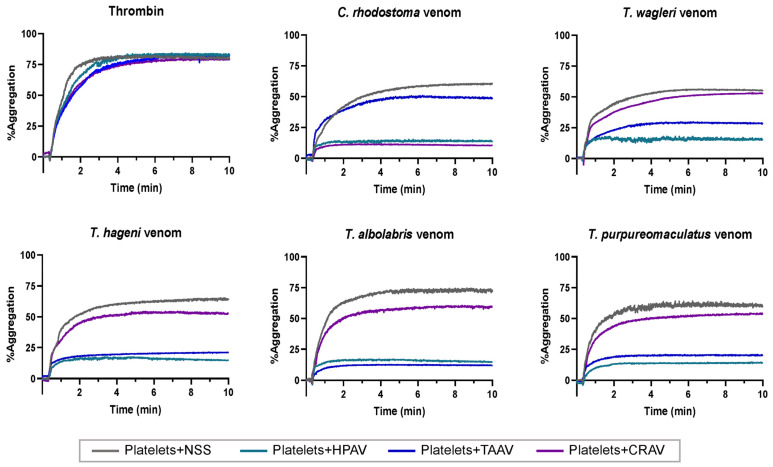
Inhibitory effect of antivenoms against venom-induced platelet aggregation. Platelet aggregation induced by thrombin and *C. rhodostoma*, *T. wagleri*, *T. hageni*, *T. albolabris*, and *T. purpureomaculatus* venoms in the presence of NSS, HPAV, TAAV, and CRAV.

**Figure 4:**
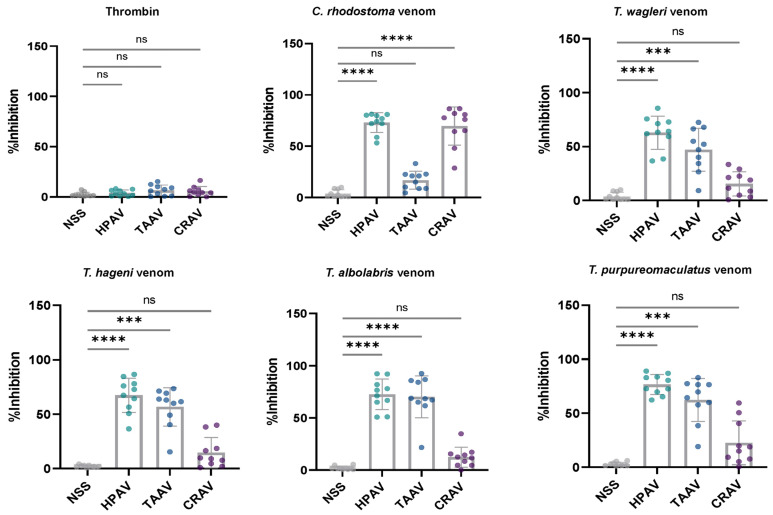
Percentage inhibition (% inhibition) of platelet aggregation induced by thrombin and *C. rhodostoma*, *T. wagleri*, *T. hageni*, *T. albolabris*, and *T. purpureomaculatus* venoms in the presence of NSS, HPAV, TAAV, and CRAV. Statistical significance was determined using the Kruskal–Wallis test followed by Dunn’s post hoc test: *** represents a *p*-value < 0.001; **** represents a *p*-value < 0.0001; ns indicates not significant.

The inhibitory effects of the antivenoms on venom-induced platelet aggregation and the % inhibition of platelet aggregation by each antivenom against the tested venoms are shown in Figure 3 and Figure 4, respectively.

Washed platelets with NSS, yielding a median %inhibition of 1.9%, served as the control. In addition, thrombin (10 IU/mL) was used as a positive control to verify the functional integrity of the washed platelets and to confirm that the antivenoms had no direct inhibitory effects on human platelet function, as no significant differences were observed between the control and antivenom-treated samples (*p* > 0.05, Figure 3 and Figure 4).

While HPAV significantly inhibited the platelet aggregation effects induced by all tested pit viper venoms, showing the highest % inhibition across all groups, CRAV exhibited a significant neutralizing effect only against *C. rhodostoma* venom-induced platelet aggregation compared to the NSS control (*p* < 0.0001, Mann–Whitney U test), but not against aggregation caused by *T. wagleri*, *T. hageni*, *T. albolabris*, or *T. purpureomaculatus* venoms (Figure 3 and Figure 4). Similarly, TAAV demonstrated a significant inhibitory effect on platelet aggregation induced by *T. albolabris* venom compared to the control (*p* < 0.0001, Mann–Whitney U test). Notably, TAAV also displayed significant cross-neutralizing effects against the venom-induced platelet aggregation activities of *T. wagleri*, *T. hageni*, and *T. purpureomaculatus* (Figure 3 and Figure 4).

### 3.4. Evaluation of Snake Venom Effects on Human Coagulation Using Venom-Induced Coagulation Test

**Figure 5:**
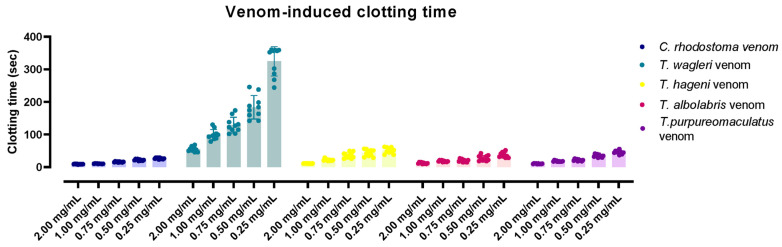
Clotting times induced by *C. rhodostoma*, *T. wagleri*, *T. hageni*, *T. albolabris*, and *T. purpureomaculatus* venoms at varying concentrations (0.25–2.00 mg/mL).

Venom-induced coagulation was assessed using various venom concentrations: 0.25, 0.50, 0.75, 1.00 and 2.00 mg/mL. The results are shown in Figure 5. The CT was observed to vary in a concentration-dependent manner across different venoms. At the highest concentration (2.00 mg/mL), *C. rhodostoma* venom demonstrated the strongest procoagulant activity, with a mean ± SD CT of 9.1 ± 0.6 s, whereas *T. wagleri* venom showed the weakest activity, with a CT of 54.6 ± 7.5 s.

Although 2.0 mg/mL resulted in the shortest CT for all venoms tested, a concentration of 1.0 mg/mL was selected for subsequent neutralizing assays to accommodate volume limitations and to maintain a consistent plasma-to-antivenom ratio of 1:1.

### 3.5. Inhibitory Effect of Antivenoms Against Venom-Induced Coagulation

**Figure 6:**
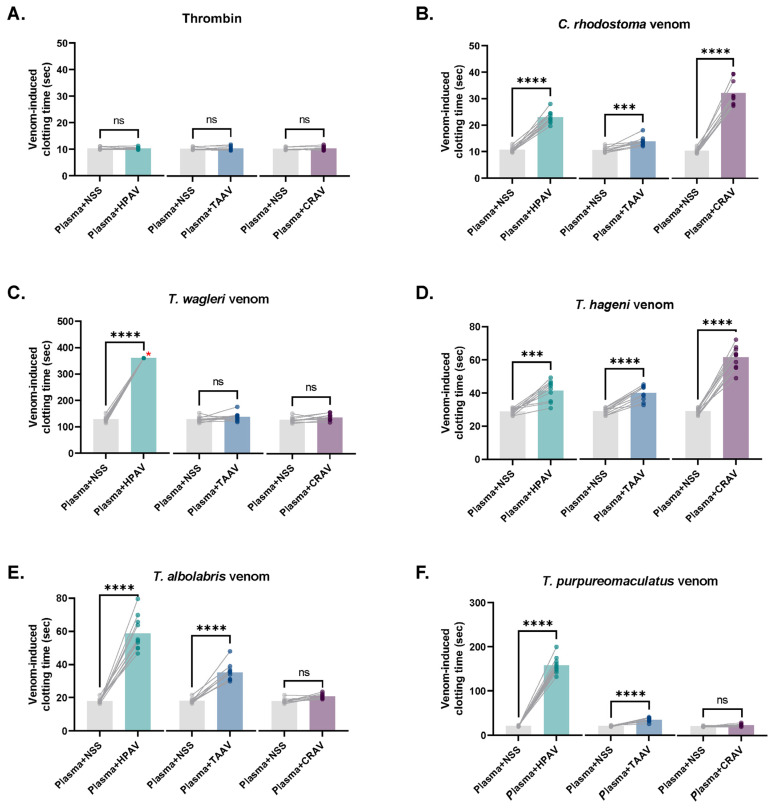
Inhibitory effect of antivenoms against venom-induced coagulation. Bar charts show clotting times (CTs) of plasma treated with NSS (control) and antivenoms (HPAV, TAAV, and CRAV) for (**A**) thrombin and (**B**) *C. rhodostoma*, (**C**) *T. wagleri*, (**D**) *T. hageni*, (**E**) *T. albolabris* and (**F**) *T. purpureomaculatus* venoms. *** represents a *p*-value < 0.001; **** represents a *p*-value < 0.0001; ns indicates not significant (paired *t*-test). Red asterisks (*) indicate unmeasurable clotting times (CT > 360.0 s), which were considered indicative of no coagulation.

**Figure 7:**
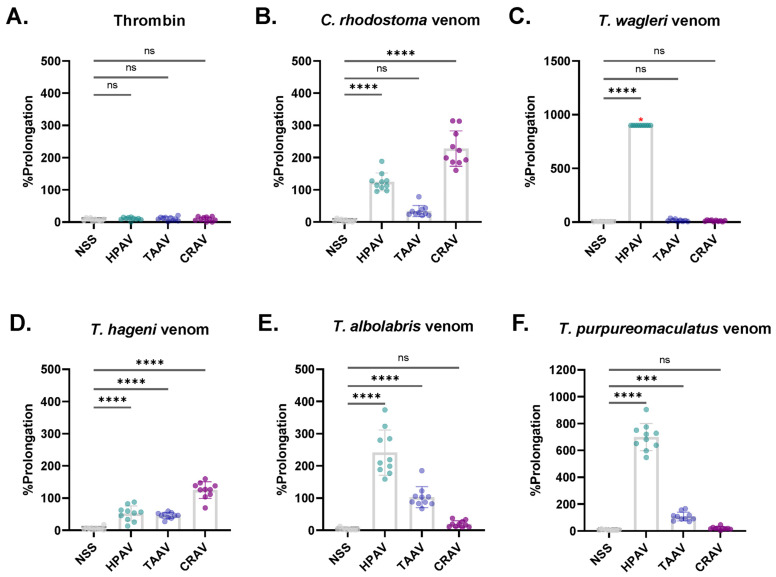
The percentage prolongation (%prolongation) of clotting time induced by (**A**) thrombin and venoms from (**B**) *C. rhodostoma*, (**C**) *T. wagleri*, (**D**) *T. hageni*, (**E**) *T. albolabris*, and (**F**) *T. purpureomaculatus* was evaluated in the presence of NSS or antivenoms: HPAV, TAAV, and CRAV. Data are expressed as median (IQR), with individual data points shown. Statistical significance was determined using one-way ANOVA followed by Tukey’s post hoc test: *** represents a *p*-value < 0.001; **** represents a *p*-value < 0.0001; ns indicates not significant. Red asterisks (*) indicate unmeasurable clotting times (CT > 360.0 s), which were considered indicative of no coagulation.

The inhibitory effects of the antivenoms on venom-induced coagulation, as well as the %prolongation of venom-induced CT by each antivenom, are presented in Figure 6 and Figure 7, respectively. The CTs of control and antivenom-treated plasma samples were compared individually (Figure 6). Thrombin (10 IU/mL) was used as a control to confirm plasma functionality and to verify that the antivenoms did not interfere with normal coagulation. No significant differences were observed between control and antivenom-treated samples (all *p* > 0.05; Figure 6A). Similarly, when %prolongation values were calculated, no significant prolongation was observed among plasma samples treated with NSS compared with baseline CTs induced by thrombin (all *p* > 0.05; Figure 7A).

Consistent with their inhibitory effects on venom-induced platelet aggregation, HPAV significantly inhibited coagulation induced by all tested pit viper venoms (all *p* < 0.001; Figure 6B–F). The mean (± SD) %prolongation values were 124.6 ± 27.8%, 52.2 ± 23.3%, 241.3 ± 70.4%, 699.4 ± 100.8%, and >900% for *C. rhodostoma*, *T. hageni*, *T. albolabris*, *T. purpureomaculatus*, and *T. wagleri* venoms, respectively (Figure 7B–F). While TAAV and CRAV did not exhibit any neutralizing effect against *T. wagleri* venom-induced coagulation, HPAV achieved complete inhibition, as evidenced by CTs exceeding 360.0 s and >900% prolongation in the coagulating effect of *T. wagleri* venom (Figure 6C and Figure 7C). Furthermore, HPAV demonstrated significantly greater neutralizing effects than TAAV against coagulation induced by *C. rhodostoma*, *T. albolabris*, and *T. purpureomaculatus* venoms (all *p* < 0.05).

As expected, CRAV exhibited a strong inhibitory effect against *C. rhodostoma* venom-induced coagulation (Figure 6B), with a mean %prolongation of 228.0% (Figure 7B). In addition, CRAV significantly prolonged the CTs induced by *T. hageni* venom (Figure 6D), with a mean %prolongation of 124.8% (Figure 7D). Interestingly, the neutralizing effects of CRAV on *C. rhodostoma* and *T. hageni* venom-induced coagulation were significantly greater than those of HPAV, with mean %prolongation values of 228.0% vs. 124.6% (*p* < 0.0001, one-way ANOVA) and 124.8% vs. 52.2% (*p* < 0.0001, one-way ANOVA), respectively.

The green pit viper monovalent antivenom (TAAV) exhibited significant neutralizing effects against the coagulation induced by *C. rhodostoma*, *T. hageni*, *T. albolabris*, and *T. purpureomaculatus* venoms, with mean %prolongation values of 34.2%, 46.1%, 102.9%, and 106.9%, respectively (all *p* < 0.001 compared to control), while showing no inhibitory effect on clotting induced by *T. wagleri* venom (Figure 6 and Figure 7B–F). Compared with HPAV, the monovalent TAAV displayed comparable inhibitory activity against *T. hageni* venom-induced coagulation but demonstrated lower neutralizing efficacy against *C. rhodostoma*, *T. albolabris*, and *T. purpureomaculatus* venoms (Figure 6 and Figure 7B–F).

## 4. Discussion

Snakebite envenoming is a major public health problem that predominantly affects rural populations worldwide, particularly in Sub-Saharan Africa, South America, South Asia, and Southeast Asia [4]. In 2017, the World Health Organization (WHO) recognized snakebite envenoming as a high-priority neglected tropical disease due to its substantial morbidity and mortality, and set a global goal to reduce snakebite-related death and disability by 50% by 2030 [26].

Appropriate treatment for snakebite envenomation is essential for optimizing patient outcomes and reducing both morbidity and mortality [6]. Venom-induced consumption coagulopathy (VICC) is the most common systemic coagulopathy associated with viperid and some elapid bites and can lead to life-threatening hemorrhage [27,28]. Asiatic pit vipers, especially *Trimeresurus* spp. and *Calloselasma* spp., are recognized for their hemotoxins, which are mostly due to the presence of thrombin-like enzyme substances [29]. Envenoming by these snakes may cause uncontrolled coagulation, which may progress to hypovolemic shock and death [30,31]. Intravenous administration of antivenom is the only effective treatment for snakebite-envenomed victims; its efficacy varies across different species and populations. This is due to the significant interspecific variation found in the toxic components of snake venoms [32]. Previous studies also reported that antivenom from one region can often display cross-reactivity and neutralize venom toxicities from similar species found in other regions as they contain a similarity in venom components [33,34]. Our RP-HPLC analysis revealed that the venom profiles of three *Trimeresurus species* (*T. albolabris*, *T. pupuremaculatus*, and *T. hageni*) were closely aligned. In contrast, the venoms of *C. rhodostoma* and *T. wagleri* showed distinct variations. These findings indicated a compositional similarity among arboreal viper venoms.

The venom components of these pit vipers contain SVMPs and SVSPs that induce several coagulation-related mechanisms, including thrombin-like activity, fibrinolysis, and platelet aggregation [10,35,36]. Several proteomic studies have reported that SVMPs, PLA_2_, and SVSPs are the most abundant protein families in the venoms of *T. albolabris*, *T. purpureomaculatus*, *T. hageni*, and *C. rhodostoma* [37,38,39]. In the present study, we evaluated the hematotoxic effects of the selected pit viper venoms on both primary and secondary hemostasis using platelet aggregation and coagulation assays, respectively. In addition, the cross-neutralizing capacity and efficacy of three commercially available snake antivenoms, TAAV, CRAV, and HPAV, were assessed.

In this study, all snake venoms effectively induced in vitro platelet aggregation, with %Max exceeding 50%. At an equal venom concentration, *T. albolabris* venom produced the highest aggregation, followed by *C. rhodostoma*, *T. purpureomaculatus*, and *T. hageni*, whereas *T. wagleri* induced the least platelet aggregation. All venoms also promoted in vitro coagulation and displayed venom concentration-dependent coagulation. At the same concentration (0.25–2.0 mg/mL), *C. rhodostoma* venom exhibited the highest procoagulant effect, whereas *T. wagleri* venom produced the longest CT, exceeding 100 s. These findings suggest a relatively weak coagulopathic effect and thrombin-like enzyme activity in *T. wagleri* venom, consistent with previous reports [40]. Unlike other Asiatic pit vipers, envenoming by *T. wagleri* typically causes localized symptoms and lacks significant hemorrhagic or coagulopathic effects [14]. In contrast, its venom is predominantly neurotoxic, mediated by peptides known as waglerins [41], while severe systemic effects are rare. A previous proteomic study demonstrated that SVMPs and SVSPs predominated in *T. albolabris* venom, whereas LAAOs and SVSPs were the most abundant protein families in Thai *T. wagleri* venom [11]. Since SVMPs are key proteins that influence the blood coagulation cascade and platelet aggregation [10], their low abundance in *T. wagleri* venom supports the weak coagulopathic effects observed for this species. Further purification and characterization of hematotoxins from *T. wagleri* venom are needed to provide insights into the mechanisms underlying its effects on hemostasis following envenomation.

Administration of specific snake antivenom is the mainstay of snakebite envenoming treatment and recommended as soon as possible in order to minimize the severity of toxic outcomes [42,43]. Clinically, polyvalent antivenoms are often considered advantageous because they reduce the risk of inappropriate antivenom use resulting from diagnosis uncertainty [44]. However, their broader antigenic coverage is associated with reduced specificity against any single venom, often necessitating higher doses to achieve effective neutralization, which increases the risk of adverse reactions as well as the financial burden on patients and their families [45].

In the present study, HPAV exhibited significant inhibitory effects on both in vitro platelet aggregation and coagulation induced by all tested pit viper venoms. This effect was particularly notable against the venoms of *T. wagleri*, *T. hageni*, and *T. purpureomaculatus*, which were not part of the immunization mixture used to produce HPAV, suggesting that HPAV possesses cross-neutralizing activity against hematotoxic effects from multiple pit viper species. In addition, previous reports demonstrated in vivo and in vitro studies of cross-neutralizing activity of HPAV on several exotic viper venoms such as *Trimeresurus fucatus* and *Hypnale hypnale* [20,46]. Moreover, HPAV has also demonstrated cross-neutralizing effects against *T. purpureomaculatus* envenomation in clinical settings. [47]

Although HPAV significantly prolonged venom-induced CTs induced by all tested pit viper venoms, it displayed a least neutralizing effect on *T. hageni* venom-induced coagulation in this study. Interestingly, compared with CRAV, the inhibitory effect of HPAV on *T. hageni* venom-induced coagulation was markedly lower. In contrast, CRAV did not exhibit any inhibitory effect on the platelet aggregation activity induced by *Trimeresurus* venoms. These discrepant findings, as well as the potential cross-neutralizing activities of HPAV and CRAV against the hematotoxic effects of *T. hageni* venom, require confirmation through additional in vitro assays and further in vivo studies.

The green pit viper monovalent antivenom (TAAV) produced by the QSMI is clinically used in treatment for arboreal pit viper envenoming in Southeast Asia. This antivenom is developed against Thai white-lipped green pit viper (*T. albolabris*) venom. The cross-reactivity or hetero-specificity of the antivenom is due to the sufficient number and amount of venom proteins in the venom, serving as antigens in antivenom production, that cover most of the available toxic protein families in Asiatic *Trimeresurus* species venoms, making it effective in cross-neutralizing venoms from phylogenetically related Asiatic *Trimeresurus* species [19,37]. TAAV could potentially provide partial protection against envenoming by other-region pit vipers which may be phylogenetically distinct but have nearly similar venom protein composition.

Our findings demonstrated that TAAV effectively neutralized in vitro platelet aggregation induced by the venoms of *T. albolabris*, *T. wagleri*, *T. hageni*, and *T. purpureomaculatus*, but not that induced by *C. rhodostoma* venom. However, TAAV did not exhibit any cross-neutralizing effect on the coagulation disturbances caused by *T. wagleri* venom. As expected, TAAV significantly inhibited the coagulant activity of *T. albolabris* venom. Notably, its neutralizing effect on venom-induced coagulation was lower than that of HPAV for the venoms of *T. purpureomaculatus* and *T. albolabris*. These findings suggested that HPAV contained immunological cross-reactivity and a degree of venom neutralization against different viper venoms. However, a previous study reported that TAAV failed to cross-neutralize several *Trimeresurus* venoms, including *T. hageni* venom, in coagulation assays using both human plasma and fibrinogen [21]. Nevertheless, the efficacy of TAAV is largely attributed to its cross-neutralization of the procoagulant toxins in various arboreal pit viper venoms, as demonstrated in in vitro antigen–antibody binding assays such as ELISA and Western immunoblotting [20,48]. Further in vivo studies on the cross-neutralizing effects of monovalent antivenoms against hematotoxic outcomes following Asian pit viper envenoming are needed as supporting evidence for its clinical use in treating Asian pit viper envenoming.

Despite providing valuable insights into the hematotoxic effects of pit viper venoms and the cross-neutralizing potential of commercially available antivenoms, this study has several limitations. First, most experiments were conducted in vitro, which may not fully capture the complexity of venom–antivenom interactions in vivo. Additional in vitro approaches, such as endothelial cytotoxicity assays and metalloproteinase inhibition tests, are recommended to further investigate the underlying mechanisms of venom-induced vascular and hemostatic dysfunction. Second, only a subset of pit viper venoms and three antivenoms were evaluated, limiting the generalizability of the findings to other regional venoms or antivenoms. Third, comprehensive dose–response analyses and in vivo validation were not performed, which are essential to confirm the clinical relevance and efficacy of the antivenoms. Additionally, geographic and seasonal variations in venom composition may affect reproducibility, and the molecular mechanisms underlying cross-neutralization were not fully explored. Future studies including a broader range of venoms from various snake species and additional antivenoms, along with in vivo validation, are recommended to address these limitations.

## 5. Conclusions

In conclusion, this study highlights the clinically relevant hematotoxic effects of Asian pit viper venoms, which impair both primary and secondary hemostasis in human platelets and plasma in vitro, underscoring the need for effective antivenom strategies. Evaluation of the cross-neutralizing capacity of monovalent and hematotoxic polyvalent antivenoms revealed the limitations of monospecific antivenoms against venoms from different snake species. Administration of polyvalent antivenoms is likely to achieve broader and more effective neutralization compared with monospecific antivenoms. These findings provide additional evidence to support healthcare providers in the management of snakebite envenoming, particularly in remote and rural areas.

## Data Availability

The original contributions presented in this study are included in the article. Further inquiries can be directed to the corresponding author.

## Abbreviations

The following abbreviations are used in this manuscript:

CRAV	*Calloselasma rhodostoma* antivenom
CT	Clotting time
ELISA	Enzyme-linked immunosorbent assay
HPAV	Hemato polyvalent antivenom
LAAO	L-amino acid oxidase
LTA	Light transmission aggregometry
PLA_2_	Phospholipase A_2_
QSMI	Queen Saovabha Memorial Institute
SVMPs	Snake venom metalloproteinases
SVSPs	Snake venom serine proteases
TAAV	*Trimeresurus albolabris* antivenom
VICC	Venom-induced consumption coagulopathy
